# Breaking stigma, discrimination and promoting rights: global evaluation of the World Health Organization QualityRights e-training on mental health, recovery and community inclusion

**DOI:** 10.1192/bjo.2025.10779

**Published:** 2025-08-18

**Authors:** Michelle Funk, Natalie Drew Bold, Charity Muturi, Ledia Lazeri, Olga Kalina, Carmen Martinez-Viciana, Debra Machando, Mauro Giovanni Carta, Gemma F. Parojinog, Simon Njuguna Kahonge, Sebnem Avşar Kurnaz, Melita Murko, Celline Cole, Layal Al Hanna, Emily McLoughlin, Magdalena Casamitjana Aguilà, Akwasi Osei, Jacob Shamuyarira, Alessandra Perra, Guadalupe Morales Cano, Zvjezdana Stjepanović, Simon Vasseur Bacle, Slađana Štrkalj-Ivezić, Ivana Svobodová, Gerli Sirk, Maria Francesca Moro

**Affiliations:** Policy, Law and Human Rights Unit, Department of Mental Health, Brain Health and Substance Use, World Health Organization, Geneva, Switzerland; Tunawiri Community Based Organisation, Nairobi, Kenya; World Health Organization Regional Office for Europe, Copenhagen, Denmark; European Network of (ex-)Users and Survivors of Psychiatry, Tbilisi, Georgia; Pan-American Health Organization/World Health Organization Regional Office for the Americas, Washington, DC, USA; World Health Organization Zimbabwe Country Office, Harare, Zimbabwe; Department of Medical Sciences and Public Health, University of Cagliari, Cagliari, Italy; Policy Linkages Office, Commission on Human Rights of the Philippines, Manila, The Philippines; Department of Mental Health, Ministry of Health, Nairobi, Kenya; World Health Organization Turkey Country Office, Ankara, Turkey; National Mental Health Programme, Ministry of Public Health, Beirut, Lebanon; Department of the Presidency, Government of Catalonia, Barcelona, Spain; Mental Health Authority, Ministry of Health, Accra, Ghana; Pamumvuri PVO, Harare, Zimbabwe; European Network of (ex-)Users and Survivors of Psychiatry, Madrid, Spain; Institute for Population and Development, Banja Luka, Bosnia and Herzegovina; WHO Collaborating Centre for Research and Training in Mental Health, Lille, France; Research Department, University Psychiatric Hospital Vrapce, Zagreb, Croatia; Health Care Department, Ministry of Health, Prague, Czechia; World Health Organization Estonia Country Office, Tallinn, Estonia

**Keywords:** WHO QualityRights, human rights, mental health, e-training, UN CRPD

## Abstract

**Background:**

There is an urgent need to address the poor quality of mental healthcare and human rights violations within mental health systems and communities. To achieve this, efforts must focus on changing the attitudes that perpetuate stigma and discrimination against individuals with mental health conditions, as well as psychosocial, intellectual and cognitive disabilities. The World Health Organization (WHO) QualityRights e-training on mental health, recovery and community inclusion is tackling these issues in several countries; however, its global impact has yet to be evaluated.

**Aims:**

This study aims to assess the changes in attitudes following the completion of the WHO QualityRights e-training in countries worldwide.

**Method:**

Data from 3026 participants were analysed in this pre-post intervention study. Changes in scores on the WHO QualityRights Attitudes questionnaire were evaluated with the paired *t*-test and Wilcoxon signed-rank test.

**Results:**

The mean differences from baseline to post-training on the WHO QualityRights Attitudes questionnaire were 9.91 (95% CI 9.58–10.24, *d* = 1.07) for the total sample, 8.95 (95% CI 8.59–9.31, *d* = 0.99) for the high-income countries sample; and 12.75 (95% CI 12.03–13.47, *d* = 1.33) for the low- and middle-income countries sample. These findings indicate that participants, after completing the e-training, showed a decrease in negative attitudes toward individuals with mental health conditions and psychosocial, intellectual and cognitive disabilities.

**Conclusions:**

This study suggests that the WHO QualityRights e-training has a positive, large effect in reducing negative attitudes toward individuals with mental health conditions and psychosocial, intellectual and cognitive disabilities, and can contribute to reduced stigma and greater alignment with rights-based approaches. These findings support the scale-up of the WHO QualityRights e-training programme.

In countries around the world, many persons with mental health conditions and psychosocial, intellectual and cognitive disabilities experience poor quality mental health services and dehumanising and degrading treatment both within the mental health system and in their communities.^1–3^ These human rights violations occur in all countries, although most of the academic research in this field has been carried out in high-income countries.^1,3^ In middle- and low-income countries, our knowledge about this issue is more fragmentary, and comes mainly from the media and reports from human rights and other non-governmental organisations.^1,4–9^ A significant challenge in mental health services is institutionalisation and the persistence of institutional cultures, even within community-based settings. Institutionalisation and associated cultures often enforce rigid routines, prioritise control over care and erode personal autonomy, ultimately leading to poor living conditions, neglect, abuse and even violence.^1,3^ Another pressing issue is the widespread use of coercive practices, and the narrow approach to treatment, care and support, that places too much emphasis on the use of medication to manage symptoms rather than taking a more holistic, rights-based and recovery approach to providing care and support.^1,2,10,11^ In many mental health facilities in low-, middle- and high-income countries, the use of seclusion and restraints remain common practices, often employed as punishment.^1–3,12^ Neglect is another pervasive yet overlooked violation, with people often left without adequate food, water or medical attention, and experiencing emotional abandonment.^13,14^ Beyond mental health facilities, people with mental health conditions and psychosocial disabilities face violence, abuse and neglect across various settings. In several low- and middle-income countries, it is common for people to be taken to traditional or faith-based healing institutions (so-called ‘prayer camps’), where they are subject to beatings, shackles and food deprivation as so-called ‘treatments’.^4^ In high-income countries, the situation is also serious with many experiencing high rates of domestic violence, often at the hands of family members or acquaintances.^15^ They are 2.5 times more likely to be assaulted or raped and five times more likely to be victims of homicide than the general population.^16,17^ Discrimination within the criminal justice system further compounds their vulnerability, as they are often not believed by police officers, and crimes against them frequently go unpunished.^18^ Furthermore, in many countries, people with mental health conditions and psychosocial, intellectual and cognitive disabilities are often denied their right to legal capacity and to decide about their treatment and other aspects of their lives,^1,19^ a practice increasingly recognised as a human rights violation since the ratification of the United Nations Convention on the Rights of Persons with Disabilities (UN CRPD).^20^ These extensive violations and resulting trauma have enduring consequences for individuals and communities.^1,2,21^

## International calls for action

International human rights standards recognise these challenges and call for major reforms in the mental health field. Four recent United Nations Human Rights Council resolutions call for a human rights approach in mental health aligned with the UN CRPD.^22–25^ These calls for action are further reinforced by the United Nations General Assembly resolution on mental health and psychosocial support^26^ and the World Health Organization (WHO)’s World Mental Health Report,^2^ which emphasise the need for countries to put in place person-centred, human rights-based, recovery-oriented mental healthcare in line with the UN CRPD. A recent joint publication by the WHO and World Psychiatric Association echoed these efforts by calling upon stakeholders in countries to take concrete action in the mental health field at policy, law, service and clinical practice levels to promote non-coercive practices and a human rights approach in mental health;^27^ for example, by using the extensive guidance developed through the WHO QualityRights initiative, which aims to promote quality of care and human rights in mental health.^28,29^

These calls for action underscore the urgent need to address poor quality mental healthcare and human rights violations within mental health systems and communities through reform efforts across several critical areas. For this to happen, a focused effort is needed to transform the attitudes and mindsets that lead to stigma and discrimination against persons with mental health conditions and psychosocial, intellectual and cognitive disabilities, which, in turn, act as barriers to put a stop to the violation of their rights. As highlighted in the 2022 Lancet Commission on this subject, stigma and discrimination that many people experience on the basis of their diagnosis of a mental health condition or intellectual and cognitive impairment marginalise and exclude them, denying them opportunities for employment, education, social protection and housing, and thus hindering their full and meaningful participation to society.^30^ To undertake reform in the mental health sector at all levels – policy, law, service and practice – it is crucial for all stakeholder groups to have compatible mindsets along with the necessary knowledge and skills to implement a human rights-based approach in mental health and challenge stigma and discrimination. Resistance to such approaches persist among many stakeholders, requiring a concerted effort to address societal misconceptions, fears and ableist views relating to mental health conditions or psychosocial, intellectual and cognitive disabilities.

## WHO QualityRights initiative

Each of these areas (policy, law, service and practice) is being addressed as part of the WHO QualityRights initiative, which aims to promote quality of care and human rights in mental health.^28^ Ending stigma and discrimination towards people with mental health conditions and psychosocial, intellectual and cognitive disabilities is at the core of this work. Many countries have been pursuing this objective for some time. However, the WHO QualityRights initiative proposes an even deeper shift in attitudes, toward recognising people with mental health conditions and psychosocial, intellectual and cognitive disabilities as rights holders and embracing their full and equal access to all human rights, including their right to full participation and inclusion as well as their right to legal capacity and freedom from forced admission or treatment and seclusion and restraints.

Within this context, the WHO has developed the QualityRights e-training on mental health, recovery and community inclusion.^31^ This e-training is based on the WHO QualityRights face-to-face capacity-building materials, but, as an online course, has the potential to have an impact on a massive global scale within and across countries. Currently, the WHO is implementing the QualityRights e-training in several countries.^32^ The research results from Ghana show substantial and significant improvements in participants’ attitudes after completion of the e-training;^33^ however, the effect of the e-training has not been evaluated on a global scale.

The present study aims to fill this gap by assessing the changes in attitudes following the completion of the WHO QualityRights e-training in countries across the world.

## Method

### Study design and setting

This naturalistic pre-post intervention study is part of a global initiative called ‘The WHO QualityRights Initiative’,^34^ launched by the WHO in 2015 to improve the quality of care in mental health and related services, and promote the rights of persons with mental health conditions and psychosocial, intellectual and cognitive disabilities.

The study participants consisted of people from a wide range of countries, who undertook the WHO QualityRights e-training (i.e. the intervention under examination) between 1 January 2022 and 31 December 2024, and completed both the pre- and post-training questionnaires.

Completing these questionnaires was not a prerequisite for participating in the e-training or receiving a certificate of completion. Participants could choose to skip them, as our primary focus was on delivering the e-training rather than collecting data for research. However, since the impact of e-learning courses is rarely evaluated, we included the questionnaire as an optional component to assess its effect. The pre- and post-training questionnaires were hosted on a separate platform, independent of the e-training platform, meaning they were not directly linked to users’ accounts. Matching responses was possible only for participants who completed both questionnaires and provided the same email address in each.

Participants were mental health and other healthcare professionals, individuals with mental health conditions and psychosocial, intellectual and cognitive disabilities, their family members or caregivers, persons with other disabilities, non-governmental organisation (NGOs) members, members of organisations for persons with disabilities and other stakeholders in the broad community; anyone with an interest was encouraged to sign up for the e-training.

### Intervention

The WHO QualityRights e-training on mental health, recovery and community inclusion aims to improve knowledge, attitudes and practices toward rights-based approaches in mental health and related services, and promote the rights of people with mental health conditions and psychosocial, intellectual and cognitive disabilities.^31^ Unlike previous training courses that focus on specific areas such as addressing stigma, promoting recovery or educating about human rights, this e-training provides a comprehensive and integrated approach covering multiple key aspects of mental health and human rights-based care and support.

At the time of this study, the e-training was available in 13 languages (Armenian, Bosnian, Croatian, Czech, English, Estonian, Filipino, French, Italian, Polish, Spanish, Turkish, Ukrainian) and covered the following topics: tackling stigma, discrimination, abuse and coercion in mental health services and the community; ensuring respect for legal capacity; taking action in support of transformation of mental health services toward a person-centred, rights-based recovery approach; taking care of one’s own mental health; and supporting friends, family and colleagues with their mental health. Although the modules are not culturally adapted for use in specific countries, they incorporate presentations, videos, interactive exercises and forum discussions that reflect diverse cultural contexts and include examples from different countries. Additionally, when the e-training is launched in a specific country, local trainers are engaged to moderate the forum discussions and ensure particular attention is given to issues relevant to the local context.

The e-training can be completed in around 24 hours and has been developed for a wide variety of stakeholders, including policy makers engaged in mental healthcare provision, health and mental healthcare providers, and persons who have received or are receiving support for their mental health.

In contrast to the past, when courses to challenge stigma and discrimination were mainly developed by health and mental health professionals, the QualityRights e-training was co-developed by a range of different stakeholders, as requested now by a growing consensus at the international level. People with mental health conditions and psychosocial, intellectual and cognitive disabilities had a pivotal role in the development of the training. Furthermore, they also have an important role in the delivery of the e-training, including through sharing their lived experience in videos and learning materials and engaging with learners in the dedicated forum.

The WHO QualityRights e-training can be accessed at ref ^35^.

### Ethics

The authors assert that all procedures contributing to this work comply with the ethical standards of the relevant national and institutional committees on human experimentation and with the Helsinki Declaration of 1975, as revised in 2013. The study gained ethical approval exemption from the World Health Organization Research Ethics Review Committee.

Participants were presented with a consent form on the WHO QualityRights e-training platform, which included details on the scope of the e-training and informed the participants that their participation was voluntary, and they could interrupt their training or data collection at any point.

### Measures

An online self-administered questionnaire was used for data collection pre- and post-training. The questionnaire included sociodemographic variables (gender, age, country, background/experience and affiliation) and the WHO QualityRights Attitudes Questionnaire (WHO QR Attitudes).^36^ The WHO QR Attitudes is a 17-item instrument to evaluate the attitudes toward people with mental health conditions and psychosocial, intellectual and cognitive disabilities as rights holders. It comprises three subscales: subscale 1, on the attitudes toward a person-centred recovery service approach; subscale 2, on the attitudes toward involuntary and coercive practices; and subscale 3, on the attitudes toward people with psychosocial disabilities or mental health conditions as decision makers and full members of society. Answers are provided on a five-point Likert scale (strongly disagree, disagree, neutral, agree and strongly agree). Scores on the questionnaire range between 17 and 85. The psychometric properties of the WHO QR Attitudes (English version) have been evaluated in a previous study, showing the instrument is valid and reliable (Cronbach’s *α* = 0.86 for the total scale, 0.61 for subscale 1, 0.77 for subscale 2 and 0.75 for subscale 3).^36^ In the present study, the instrument showed good internal consistency (Cronbach’s *α* = 0.85 for the total scale, 0.68 for subscale 1, 0.72 for subscale 2 and 0.74 for subscale 3).

### Analysis

First, respondents who did provide informed consent but did not continue the pre- or post-training questionnaire were excluded. Second, respondents who did not complete the WHO QR Attitudes questionnaire either at the pre- or post-training questionnaire were removed. Third, respondents who completed the pre- and post-training questionnaire were matched by their email address.

Descriptive analyses were conducted for the sociodemographic variables. Univariate analyses were used to examine continuous variables by assessing the mean, median and spread of the data. Tabular analyses were used to examine categorical variables by assessing frequencies. Missing data were reported for each variable.

The categorical variable ‘country’ was recoded to create two new variables: region’ (based on countries belonging to the list of WHO regions)^37^ and income (based on the World Bank classification).^38^

Potential differences between participants who completed the pre-training questionnaire (and thus started the e-training) and participants in the matched sample were reported. The completion rate for the e-training was calculated as the proportion of participants who completed the e-training out of the total number of participants who started it between 1 January 2022 and 31 December 2024.

Only the matched sample was used to evaluate changes in attitudes. Data analyses (paired *t*-test for normally distributed data and Wilcoxon signed-rank test for non-normally distributed data) were conducted to evaluate the change in the scores at the WHO QR Attitudes (total and subscales) in the overall sample and in participants from high-income and low- and middle-income countries after completing the QualityRights e-training. The analysis of variance (ANOVA) test was performed to compare changes in the total scores at the WHO QR Attitudes in high-income versus low- and middle-income countries. Similar analyses were conducted for the two largest participant categories: health practitioners and mental health or related practitioners. For normally distributed data, we reported effect sizes as mean differences with 95% confidence intervals (and, additionally, as Cohen’s *d* and Hedges’ *g* approximation: *d* = 0.2, 0.5 and 0.8 were considered as small, medium and large effects, as per convention).^39^ For non-normally distributed data, we reported test statistics and *P*-values. Analyses were performed using SAS (version 9.4 for Windows; SAS Institue Inc., Cary, North Carolina, USA; https://www.sas.com/) and R (version 4.4.1 for macOS; The R Project for Statistical Computing, Auckland, New Zealand; https://cran.rproject.org/bin/macosx/base/R4.4.1.pkg). Figures were created using Microsoft Excel (version 16.86 for macOS) and R (version 4.4.1 for macOS; ggplot2 package; The R Project for Statistical Computing, Auckland, New Zealand; https://cran.rproject.org/package=ggplot2).

## Results

Between 1 January 2022 and 31 December 2024, a total of 77 832 participants enrolled in the e-training (27 968 from high-income countries, 49 787 from low- and middle-income countries and 77 with no country data). Of these, 42 162 participants (54.17%) completed the training (13 026 from high-income countries, 29 122 from low- and middle-income countries and 14 with no country data) (Supplementary Material 2 available online at https://doi.org/10.1192/bjo.2025.10779). Completion rates were 46.58% in high-income countries and 58.49% in low- and middle-income countries.

Overall, a total of 26 702 participants began the pre-training questionnaire, with 21 363 completing it. A total of 10 485 participants began the post-training questionnaire, with 6535 completing it. It was possible to match 3026 participants.

**Fig. 1:**
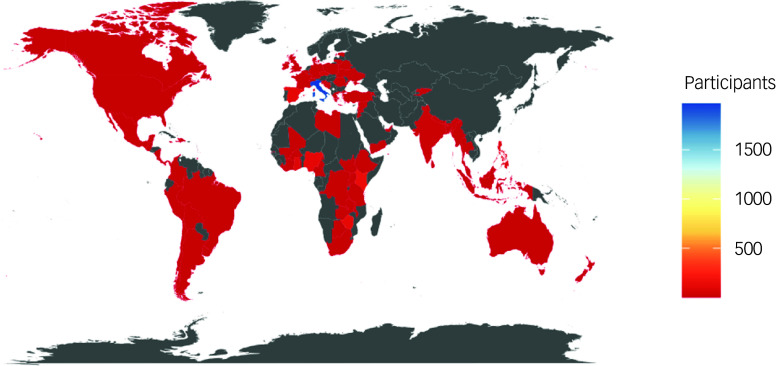
Map of participants by country (matched sample, *n* = 3026; years 2022–2024).

Table 1 shows the characteristics of participants in the matched sample (used for the analyses). Overall, 72.33% of participants identified as women, 26.63% as men and 0.94% as other gender. The median age was 43 years. With respect to the country of residence, 74.10% of the participants resided in a high-income country and 25.90% in a low- and middle-income country; with 73.63% of the participants reporting that they resided in the WHO European Region and 17.28% in the WHO African Region (Fig. 1). Most participants were health practitioners (46.03%) or mental health or related practitioners (25.21%). More than half of the participants worked either in a general health service (34.60%) or in a mental health service (21.45%), whereas others were affiliated with a professional organisation (7.67%), a Ministry of Health (4.36%), an NGO (4.10%) or were university students (4.69%).

**Table 1:** Sociodemographic characteristics of the sample

Characteristics	Matched sample (*n* = 3026) *n* (%) or mean (s.d.)
Gender^a^	
Women	2162 (72.33)
Men	796 (26.63)
Other gender	28 (0.94)
Prefer not to answer	3 (0.10)
Age, years	
	42.43 (13.84)
Region^b^	
AFRO	521 (17.28)
EMRO	71 (2.35)
EURO	2220 (73.63)
AMRO	63 (2.09)
SEARO	35 (1.16)
WPRO	105 (3.48)
Income^b^	
High	2234 (74.10)
Low-middle	781 (25.90)
Background/experience	
Academia	150 (4.96)
Administration/management	56 (1.85)
Family member or care partner	58 (1.92)
Health practitioner	1393 (46.03)
Human rights advocate	25 (0.83)
Lawyer	14 (0.46)
Mental health or related practitioner	763 (25.21)
Person with lived experience/person with psychosocial, intellectual or cognitive disability	118 (3.90)
Person with other disabilities	46 (1.52)
Policy maker/analyst	7 (0.23)
Other	396 (13.09)
Affiliation	
Academia	60 (1.98)
Organisations of persons with disabilities	83 (2.74)
Donor/funder	1 (0.03)
General health service	1047 (34.60)
Mental health service	649 (21. 45)
Ministry of health	132 (4.36)
Multilateral organisation or development agency	4 (0.13)
Non-governmental organisations	124 (4.10)
Other government ministry/department/commission	66 (2.18)
Professional organisations/associations	232 (7.67)
Students (secondary school)	3 (0.10)
Students (university)	142 (4.69)
United Nations organisations and agencies	9 (0.30)
World Health Organization	19 (0.63)
Other	455 (15.04)

AFRO, African Region; AMRO, Region of the Americas; EMRO, Eastern Mediterranean Region; EURO, European Region; SEARO, South-East Asia Region; WPRO, Western Pacific Region.

a. Missing data (*n* = 37) for gender.

b. Some participants (*n* = 11) reported that they reside in a country not registered in the World Health Organization’s list of countries, and thus they were not classifiable by region or income.

The sociodemographic characteristics of the matched (*n* = 3026) and pre-training questionnaire responses (*n* = 21 363) are compared in Supplementary Material 1. Overall, the two samples are comparable with respect to gender and age, but present differences with respect to country, background/experience and affiliation.

### Attitude changes (WHO QR Attitudes questionnaire total score)

**Fig. 2:**
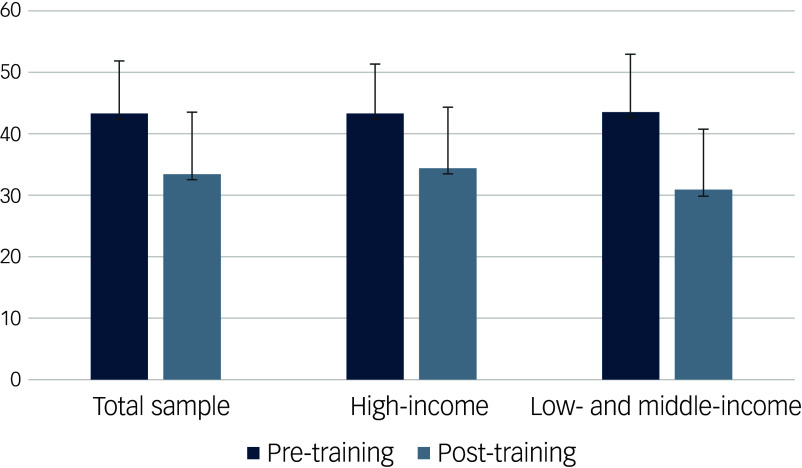
Attitudes before (pre-training) and after (follow-up) completing the WHO QualityRights e-training. Error bars indicate standard deviations. A lower score indicates less negative attitudes toward persons with mental health conditions and psychosocial, intellectual and cognitive disabilities.

On average, we found that participants in the total matched sample had lower scores (indicating less negative attitudes) at the WHO QR Attitudes questionnaire after completing the WHO QR e-training (43.51 pre-training to 33.60 post-training) (Table 2, Fig. 2). This effect was maintained when evaluating separately the high-income and the low- and middle-income countries (43.44 pre-training to 34.50 post-training and 43.69 pre-training to 30.94 post-training, respectively) (Table 2, Fig. 2).

**Table 2:** Changes in mean total scores at the WHO QualityRights (WHO QR) Attitudes pre- and post-training (total sample, high-income countries and low- and middle-income countries)

	Mean (s.d.)		Percentage improvement
WHO QR Attitudes total score	Pre-training	Post-training	
Total sample	43.51 (8.43)	33.60 (10.06)	22.78
High-income countries	43.44 (8.10)	34.50 (9.95)	20.58
Low- and middle-income countries	43.69 (9.30)	30.94 (9.86)	29.18

As reported in Table 3, Paired samples *t*-tests showed that the mean differences from baseline to post-training at the WHO QR Attitudes questionnaire had large effect sizes: the mean difference post-training was 9.91 (95% CI 9.58–10.24) for the total sample (*d* = 1.07), 8.95 (95% CI 8.59–9.31) for the high-income countries sample (*d* = 0.99) and 12.75 (95% CI 12.03–13.47) for the low- and middle-income countries sample (*d* = 1.33).

**Table 3:** Inferential statistics (paired *t*-test) on average change at the total scores at the WHO QualityRights (WHO QR) Attitudes following the e-training (total sample, high-income countries and low- and middle-income countries)

WHO QR Attitudes total score	Mean difference	95% CI	*d*^a^
Total sample	9.91	9.58–10.24	1.07
High-income countries	8.95	8.59–9.31	0.99
Low- and middle-income countries	12.75	12.03–13.47	1.33

a. Hedges’ *g*-values were nearly identical across all comparisons, with only negligible differences at higher decimal places.

These findings indicate that participants, after completing the e-training, had less negative attitudes toward persons with mental health conditions and psychosocial, intellectual and cognitive disabilities.

The effect of the e-training appeared to be larger in low- and middle-income countries compared with high-income countries (ANOVA test: mean difference 3.81, 95% CI 3.06–4.55).

With respect to the two largest participants categories, health practitioners and mental health or related practitioners, both groups showed lower scores on the WHO QR Attitudes Questionnaire after completing the e-training, indicating less negative attitudes (45.12 pre-training to 34.12 post-training for health practitioners; 39.18 pre-training to 31.22 post-training for mental health and related practitioners) (Supplementary Material 3). Paired samples *t*-tests showed that the mean differences from baseline to post-training on the WHO QR Attitudes questionnaire had large effect sizes: the mean difference post-training was 11.01 (95% CI 10.53–11.48) for the health practitioners sample (*d* = 1.27) and 7.95 (95% CI 7.37–8.54) for the mental health and related practitioners sample (*d* = 0.87) (Supplementary Material 4), The effect of the e-training appeared to be larger for health practitioners compared with mental health and related practitioners (ANOVA test: mean difference 3.05, 95% CI 2.28–3.83).

### Attitude changes (WHO QR Attitudes questionnaire subscales score)

We found that participants had lower scores on the WHO QR Attitudes questionnaire subscales after completing the WHO QR e-training (Table 4), indicating less favourable attitudes toward a mental health services approach that is not based on human rights and holistic care (subscale 1) and involuntary and coercive practices (Subscale 2), and less negative attitudes toward persons with mental health conditions and psychosocial, intellectual and cognitive disabilities as rights holders (subscale 3).

**Table 4:** Median scores pre- and post-training at the WHO QualityRights (WHO QR) Attitudes subscales (total sample, high-income countries and low- and middle-income countries)

WHO QR Attitudes subscales score^a^	First quartile, median, third quartile	
	Pre-training	Post-training
Subscale 1 Total sample	16, 19, 22	12, 16, 19
Subscale 1 high-income	16, 19, 22	13, 16, 19
Subscale 1 low- and middle-income	16, 19, 23	11, 14, 18
Subscale 2 Total sample	9, 11, 12	5, 8, 9
Subscale 2 high-income	9, 11, 12	6, 8, 9
Subscale 2 low- and middle-income	9, 11, 13	4, 7, 9
Subscale 3 Total sample	12, 14, 16	8, 10, 12
Subscale 3 high-income	12, 14, 16	8, 10, 12
Subscale 3 low- and middle-income	11, 13, 15	6, 9, 11

a. The WHO QR Attitudes questionnaire comprises three subscales: (1) attitudes toward a person-centred recovery-oriented service approach; (2) attitudes toward involuntary and coercive practices; and (3) attitudes toward people with psychosocial disabilities or mental health conditions as decision makers and full members of society.

As reported in Table 5, the Wilcoxon signed-rank tests show that differences post-training were highly statistically significant (*P* < 0.0001) on subscale 1, attitudes toward person centred-recovery service approach; subscale 2, attitudes toward involuntary and coercive practices; and subscale 3, attitudes toward people with psychosocial disabilities or mental health conditions as decision makers and full members of society. This effect was maintained when analysing the high-income and low- and middle-income countries samples separately.

**Table 5:** Inferential statistics (Wilcoxon signed-rank test) on change at the subscales scores of the WHO QualityRights (WHO QR) Attitudes following the e-training (total sample, high-income countries and low- and middle-income countries)

WHO QR Attitudes^a^	Wilcoxon signed-rank statistic	*P*-value
Subscale 1 total sample	1 403 622	<0.0001
Subscale 1 high-income	713 127	<0.0001
Subscale 1 low- and middle-income	111 351.5	<0.0001
Subscale 2 total sample	1 661 841	<0.0001
Subscale 2 high-income	874 928	<0.0001
Subscale 2 low- and middle-income	122 118	<0.0001
Subscale 3 total sample	1 720 228	<0.0001
Subscale 3 high-income	927 782.5	<0.0001
Subscale 3 low- and middle-income	119 920.5	<0.0001

a. The WHO QR Attitudes scale comprises three subscales: (1) attitudes toward a person-centred recovery-oriented service approach; (2) attitudes toward involuntary and coercive practices; and (3) attitudes toward people with psychosocial disabilities or mental health conditions as decision makers and full members of society.

Similar effects to the ones reported in Tables 4 and 5 were observed when analysing the health practitioners and mental health and related practitioners’ samples separately (Supplementary Materials 5 and 6).

## Discussion

The present study is the first to investigate the effect of the WHO QualityRights e-training on the attitudes of a large sample of stakeholders from countries across the world. Our findings indicate that the e-training has a large, positive impact in changing attitudes toward persons with mental health conditions and psychosocial, intellectual and cognitive disabilities (with a 22.78% improvement in attitudes in the overall sample). This shift can contribute to reduced stigma and greater alignment with rights-based approaches. Notably, this effect seems to be more pronounced in low- and middle-income countries than in high-income countries (29.18 *v.* 20.58% improvement in attitudes, respectively). Similar results are found when examining separately the attitudes toward a person centred-recovery service approach, involuntary and coercive practices, and people with mental health conditions and psychosocial, intellectual and cognitive disabilities as decision makers and full members of society.

These findings are aligned with those of a recent paper examining the impact of the WHO QualityRights e-training in Ghana, where authors found that completing the e-training resulted in significant attitude changes toward alignment with human rights (with approximately a 40% improvement in attitudes in the overall sample).^33^

It is interesting to note the greater impact of the e-training in low- and middle-income countries and Ghana (a low- and middle-income country itself) than in high-income countries. In our study, this finding does not seem to be driven by a different baseline score on the WHO QR Attitudes questionnaire. One explanation can be that among participants recruited in low- and middle-income countries – where internet connection has a higher cost and is not always available, and most people were not receiving incentives to complete the course – only those more interested in the topics of the e-training (and thus more prone to a change in their attitudes) completed the e-training and, additionally, the evaluation questionnaires. On the contrary, among participants recruited in high-income countries, many were receiving incentives to complete the training in the form of Continuing Professional Development (CPD) credits, and thus they could have been motivated to complete it even though they did not have a particular interest in the topic or disagreed with its contents (and thus, they were less prone to a change in their attitudes). Furthermore, since CPD credits often require completing evaluation questionnaires, participants receiving this incentive may have been more likely to fill them out, even though it was not mandatory. The greater impact of e-training on attitudes in low- and middle-income countries compared with high-income countries could also be attributed to systemic inequalities and disparities in access to resources. For instance, low- and middle-income countries may have more limited access to learning opportunities, information and insights on mental health, which could make participants more receptive to the e-training content and thus more likely to experience a shift in attitudes.

Despite these differences, the present study shows that the WHO QualityRights e-training is effective and can be a fundamental instrument to positively modify the attitudes of various stakeholders (e.g. health providers and mental health and related providers). Notably, the e-training also shows high feasibility, as reflected in its completion rates: 54.17% in the total sample, 46.58% in high-income countries and 58.49% in low- and middle-income countries. These rates are significantly higher than those typically seen in massive open online courses, where a completion rate of 10–30% is generally considered good.^40^ The strong completion rates indicate that the training successfully engages participants and can be implemented on a large scale. An online course has the potential to build capacity globally at a much lower cost and shorter period of time than is possible through face-to-face training alone. For these reasons, the WHO QualityRights e-training is a tool that can contribute to sustainable change, helping to fight stigma and discrimination and supporting the ongoing shift of mental health systems and communities toward a rights-based, person-centred approach in mental health. However, although improved knowledge and attitudes toward human rights and recovery-based approaches are crucial, they alone are not enough. Meaningful change requires translating knowledge and attitudes into practice, supported by improved availability and access to rights-based community services. Additionally, reform is unlikely to progress if only select groups of stakeholders are empowered and trained to challenge stigma and discrimination. For lasting and meaningful change, shifts in both mindsets and practices must occur at all levels of society.

With this in mind, it would be important to scale-up the WHO QualityRights e-training on a large scale, reaching as many countries as possible and large numbers of persons within communities in countries. This would help foster the societal mindset shift needed to implement the practice changes emphasised by the QualityRights e-training. Additionally, focused and strategic in-person training on QualityRights, combined with complementary strategies at the policy, legal and service levels, should be introduced to enhance the impact and ensure the sustainability of change. It would also be essential to ensure that a wide range of stakeholders (and not only, as in the past, mental and health workers) are empowered and trained to maintain this change over time. Further research should be conducted to evaluate the effect of the e-training in changing not only attitudes, but also practices to promote quality of care and human rights in mental health. In addition to quantitative methods, qualitative and mixed methods could be used to understand the impact of the QualityRights e-training.

Currently, the WHO QualityRights e-training has been made available worldwide for free, and, as such, many other countries and participants can engage in this course and acquire knowledge and skills to challenge stigma and discrimination and the status quo in mental health.

Nevertheless, there are some limitations of this study that need to be mentioned and could help new stakeholders and countries benefit from this course.

First, we used a convenience sample composed by people who undertook the WHO QualityRights e-training and completed the pre- and post-training questionnaires. Unfortunately, these questionnaires are not directly linked to the user’s account and thus it was not always possible to match the pre- and post-training questionnaires for data analysis.

In addition, the questionnaires were not mandatory, and thus many participants may have completed the e-training without any, or only a partial, evaluation of their attitudes. As a result, we were only able to analyse data from the total matched sample. This is a missed opportunity in terms of data collection and could have biased the results in addition to reducing our sample size. In the future, it would be important to include the pre- and post-training questionnaires as essential elements of the e-training platform, linked directly to the user’s account, to ensure that responses can be matched and data are not missed.

Second, certain countries were more represented than others (for instance, high-income countries represented 74.10% of the sample), and this may limit the generalisability of the findings. However, we provided separated analyses for the overall sample and the high-income and low- and middle-income countries samples to take into account this limitation, and results confirmed the impact of the e-training regardless of the country income level.

Third, certain stakeholder groups were more represented than others. For instance, mental and health practitioners represented 71.24% of the sample, whereas persons with mental health conditions and psychosocial, intellectual and cognitive disabilities only 3.90% of the sample. This may indicate either that groups other than mental and health practitioners are less likely to receive information about the training or that they are less likely to complete it. Future initiatives to scale-up the e-training should specifically target groups other than mental and health practitioners, to increase their participation. Furthermore, research should focus on evaluating the impact of the e-training in participants from these groups.

Fourth, it is possible that participants who found the training too difficult or not aligned with their beliefs may have not completed the course and thus the follow-up questionnaire. This may have biased the results away from the null, thus contributing to the large effect sizes. Social desirability bias may also have affected the result, with participants more likely (particularly after the training) to give answers that reflect what they think the researchers would like to hear, rather than their real attitudes or beliefs. Future studies may use measures of social desirability such as the Marlowe–Crowne Social Desirability Scale to evaluate if participants may have underreported negative attitudes. Furthermore, it is important to acknowledge that self-reported improvements may not always translate into real or sustained attitude change. Research should extend beyond self-reported changes in attitudes to explore objective indicators of attitude and practice shifts in routine care, providing a more comprehensive evaluation of the e-training’s real-world impact.

In conclusion, this study shows that the WHO QualityRights e-training has a positive, large effect in reducing negative attitudes towards individuals with mental health conditions and psychosocial, intellectual and cognitive disabilities, and can contribute to reduced stigma and greater alignment with rights-based approaches.

The results suggest that scaling up the QualityRights e-training could help to foster change and support the ongoing shift of mental health systems and communities toward a rights-based, person-centred approach.

## Supporting information

Funk et al. supplementary material Funk et al. supplementary material

## Data Availability

The data that support the findings of this study are available from the corresponding author, M.F., upon reasonable request.

## Supplementary material

The supplementary material is available online at https://doi.org/10.1192/bjo.2025.10779

## References

[1] Drew N , Funk M , Tang S , Lamichhane J , Chavez E , Katontoka S , et al. Human rights violations of people with mental and psychosocial disabilities: an unresolved global crisis. Lancet 2011; 378: 1664–75. 22008426 10.1016/S0140-6736(11)61458-X

[2] World Health Organization. World Mental Health Report: Transforming Mental Health for All. World Health Organization, 2022 (https://www.who.int/publications/i/item/9789240049338).

[3] Mfoafo-M’Carthy M. Human rights violations and mental illness: implications for engagement and adherence. SAGE Open 2014; 4: 1–18.

[4] Human Rights Watch. *‘Like a Death Sentence’: Abuses Against Persons with Mental Disabilities in Ghana*. Human Rights Watch, 2012 (https://www.hrw.org/report/2012/10/02/death-sentence/abuses-against-persons-mental-disabilities-ghana).

[5] Human Rights Watch. *Nigeria: People with Mental Health Conditions Chained, Abused*. Human Rights Watch, 2019 (https://www.hrw.org/news/2019/11/11/nigeria-people-mental-health-conditions-chained-abused).

[6] Human Rights Watch. *Living in Hell: Abuses Against People with Psychosocial Disabilities in Indonesia*. Human Rights Watch, 2016 (https://www.hrw.org/report/2016/03/20/living-hell/abuses-against-people-psychosocial-disabilities-indonesia).

[7] Mental Disability Rights International. *Human Rights and Mental Health in Peru*. Mental Disability Rights International, 2004 (https://www.driadvocacy.org/sites/default/files/2024-01/Peru_ENG.pdf).

[8] Moro MF , Kola L , Fadahunsi O , Jah EM , Kofie H , Samba D , et al. Quality of care and respect of human rights in mental health services in four West African countries: collaboration between the mental health leadership and advocacy programme and the World Health Organization QualityRights initiative. BJPsych Open 2022; 25: e31. 10.1192/bjo.2021.1080 PMC8811781 35076357

[9] Moro MF , Carta MG , Gyimah L , Orrell M , Amissah C , Baingana F , et al. A nationwide evaluation study of the quality of care and respect of human rights in mental health facilities in Ghana: results from the World Health Organization QualityRights initiative. BMC Public Health 2022; 22: 639. 35366832 10.1186/s12889-022-13102-2 PMC8976418

[10] Sashidharan S , Saraceno B. Is psychiatry becoming more coercive? The rising trend is damaging for patients, unsupported by evidence, and must be reversed. BMJ 2017; 357: 2904. 10.1136/bmj.j2904 28642351

[11] Savage MKLP , Newton-Howes G , Arnold R , Staggs VS , Kisely S , Hasegawa T , et al. Comparison of coercive practices in worldwide mental healthcare: overcoming difficulties resulting from variations in monitoring strategies. BJPsych Open 2024; 10: e26. 38205597 10.1192/bjo.2023.613 PMC10790218

[12] Patel V , Saxena S , Lund C , Thornicroft G , Baingana F , Bolton P , et al. The Lancet Commission on global mental health and sustainable development. Lancet 2018; 392: 1553–98. 30314863 10.1016/S0140-6736(18)31612-X

[13] Lund C. Mental health and human rights in South Africa: the hidden humanitarian crisis FOREWORD. S Afr J Hum Rights 2016; 32: 403–5.

[14] Gostin L. ‘Old’ and ‘new’ institutions for persons with mental illness: treatment, punishment or preventive confinement? Public Health 2008; 122: 906–13. 18555496 10.1016/j.puhe.2007.11.003

[15] Trevillion K , Oram S , Feder G , Howard LM. Experiences of domestic violence and mental disorders: a systematic review and meta-analysis. PLoS One 2012; 7: e51740. 23300562 10.1371/journal.pone.0051740 PMC3530507

[16] Hiday VA. Putting community risk in perspective: a look at correlations, causes and controls. Int J Law Psychiatry 2006; 29: 316–31. 16533532 10.1016/j.ijlp.2004.08.010

[17] Crump C , Sundquist K , Winkleby MA , Sundquist J. Mental disorders and vulnerability to homicidal death: Swedish nationwide cohort study. BMJ 2013; 346: f557. 23462204 10.1136/bmj.f557 PMC6364268

[18] Khalifeh H , Johnson S , Howard LM , Borschmann R , Osborn D , Dean K , et al. Violent and non-violent crime against adults with severe mental illness. Br J Psychiatry 2015; 206: 275–82. 25698767 10.1192/bjp.bp.114.147843

[19] Puras D , Gooding P. Mental health and human rights in the 21st century. World Psychiatry 2019; 18: 42–3. 30600633 10.1002/wps.20599 PMC6313250

[20] United Nations. *United Nations Convention on the Rights of Persons with Disabilities*. United Nations, 2006 (https://www.un.org/development/desa/disabilities/convention-on-the-rights-of-persons-with-disabilities/convention-on-the-rights-of-persons-with-disabilities-2.html).

[21] Chieze M , Hurst S , Kaiser S , Sentissi O. Effects of seclusion and restraint in adult psychiatry: a systematic review. Front Psychiatry 2019; 10: 491. 31404294 10.3389/fpsyt.2019.00491 PMC6673758

[22] United Nations Human Rights Council. Resolution A/HRC/32/18, Mental Health and Human Rights. United Nations Human Rights Council, 2016 (https://digitallibrary.un.org/record/845623/files/A_HRC_RES_32_18-EN.pdf).

[23] United Nations Human Rights Council. Resolution A/HRC/RES/36/13, Mental Health and Human Rights. United Nations Human Rights Council, 2017 (https://undocs.org/A/HRC/RES/36/13).

[24] United Nations Human Rights Council. Resolution A/HRC/RES/43/13, Mental Health and Human Rights. United Nations Human Rights Council, 2020 (https://undocs.org/A/HRC/RES/43/13).

[25] United Nations Human Rights Council. Resolution A/HRC/52/L.15, Mental Health and Human Rights. United Nations Human Rights Council, 2023 (https://digitallibrary.un.org/record/4013136?ln=en).

[26] United Nations General Assembly. Resolution A/RES/77/300, Mental Health and Psychosocial Support. United Nations General Assembly, 2023 (https://docs.un.org/en/A/RES/77/300).

[27] Gill N , Drew N , Rodrigues M , Muhsen H , Morales Cano G , Savage M , et al. Bringing together the World Health Organization’s QualityRights initiative and the World Psychiatric Association’s programme on implementing alternatives to coercion in mental healthcare: a common goal for action. BJPsych Open 2024; 10: e23. 38179597 10.1192/bjo.2023.622 PMC10790219

[28] World Health Organization. WHO QualityRights Materials for Training, Guidance and Transformation. World Health Organization, 2015 (https://www.who.int/mental_health/policy/quality_rights/en/). 10.1192/bjp.2021.20 33645494

[29] Funk MB , Ansong J , Chisholm D , Murko M , Nato J , Ohene SA , et al. Strategies to achieve a rights-based approach through WHO QualityRights. In Mental Health, Legal Capacity, and Human Rights (eds MM Stein , F Patel , V Sunkel ): 244–59. Cambridge University Press, 2021.

[30] Thornicroft G , Sunkel C , Alikhon Aliev A , Baker S , Brohan E , el Chammay R , et al. The Lancet Commission on ending stigma and discrimination in mental health. Lancet 2022; 400: 1438–80. 36223799 10.1016/S0140-6736(22)01470-2

[31] World Health Organization. Mental Health, Brain Health and Substance Use: WHO QualityRights e-Training on Mental Health. World Health Organization, 2017 (https://www.who.int/teams/mental-health-and-substance-use/policy-law-rights/qr-e-training).

[32] Moro MF , Pathare S , Zinkler M , Osei A , Puras D , Paccial R , et al. The WHO QualityRights initiative: building partnerships among psychiatrists, people with lived experience and other key stakeholders to improve the quality of mental healthcare. Br J Psychiatry 2022; 220: 49–51. 10.1192/bjp.2021.147 35049475

[33] Poynton-Smith E , Orrell M , Osei A , Ohene SA , Ansong J , Gyimah L , et al. A quantitative analysis of human rights-related attitude changes towards people with mental health conditions and psychosocial, intellectual, or cognitive disabilities following completion of the WHO QualityRights e-training in Ghana. Int J Ment Health Syst 2023; 17: 46. 38053116 10.1186/s13033-023-00609-3 PMC10698997

[34] Funk M , Drew N . WHO QualityRights: transforming mental health services. Lancet Psychiatry 2017; 4: 826–7. 28711282 10.1016/S2215-0366(17)30271-7

[35] World Health Organization. QualityRights Materials for Training, Guidance and Transformation. WHO, 2019 (https://www.who.int/publications/i/item/who-qualityrights-guidance-and-training-tools). 10.1192/bjp.2021.20 33645494

[36] Moro MF. Evaluating the psychometric properties of three WHO instruments to assess knowledge about human rights, attitudes toward persons with mental health conditions and psychosocial disabilities, and practices related to substitute decision-making and coercion in mental health. Front Psychiatry 2024; 15: 1435608. 39310660 10.3389/fpsyt.2024.1435608 PMC11413867

[37] World Health Organization. Countries. World Health Organization, 2017 (https://www.who.int/countries).

[38] World Bank. World Bank Country and Lending Groups. World Bank, 2023 (https://datahelpdesk.worldbank.org/knowledgebase/articles/906519-world-bank-country-and-lending-groups).

[39] Morris SB. Estimating effect sizes from pretest-posttest-control group designs. Organ Res Methods 2008; 11: 364–86.

[40] Jordan K. Massive Open Online Course completion rates revisited: assessment, length and attrition. Int Rev Res Open Distrib Learn 2015; 16: 341–58.

