# Supplementary material for: Breaking stigma, discrimination and promoting rights: global evaluation of the World Health Organization QualityRights e-training on mental health, recovery and community inclusion
Source: BJPsych Open. 2025 Aug 18;11(5):e185. doi: 10.1192/bjo.2025.10779 (PMC12451731; doi:10.1192/bjo.2025.10779)
Supplement: Funk et al. supplementary material [file S2056472425107795sup001.docx]

Supplementary Material 1: Table comparing the sociodemographic characteristics of the matched sample and the sample with participants who completed the QR Attitudes pre-training questionnaire

|  | **Matched sample**  **(n=3,026)**  **n (%) or mean (SD)** | **Pre-training sample**  **(n=21,363)**  **n (%) or mean (SD)** |
| --- | --- | --- |
| **Gender*** |  |  |
| Women | 2,162 (72.33) | 14,494 (68.42) |
| Men | 796 (26.63) | 6,414 (30.28) |
| Other gender | 28 (0.94) | 223 (1.05) |
| Prefer not to answer | 3 (0.10) | 54 (0.25) |
| **Age** |  |  |
|  | 42.43 (13.84) | 38.71 (13.53) |
| **Region**** |  |  |
| AFRO | 521 (17.28) | 6,080 (28.53) |
| EMRO | 71 (2.35) | 705 (3.31) |
| EURO | 2,220 (73.63) | 10,869 (51.01) |
| AMRO | 63 (2.09) | 1071 (5.03) |
| SEARO | 35 (1.16) | 692 (3.25) |
| WPRO | 105 (3.48) | 1,891 (8.87) |
| **Income**** |  |  |
| High | 2,234 (74.10) | 11,287 (52.98) |
| Low Middle | 781 (25.90) | 10,017 (47.02) |
| **Background/experience** |  |  |
| Academia | 150 (4.96) | 2,020 (9.46) |
| Administration/Management | 56 (1.85) | 805 (3.77) |
| Family member or care partner | 58 (1.92) | 564 (2.64) |
| Health practitioner | 1,393 (46.03) | 7,254 (33.96) |
| Human rights advocate | 25 (0.83) | 372 (1.74) |
| Lawyer | 14 (0.46) | 99 (0.46) |
| Mental health or related practitioner | 763 (25.21) | 5,910 (27.66) |
| Person with lived experience/Person with psychosocial, intellectual or cognitive disability | 118 (3.90) | 922 (4.32) |
| Person with other disabilities | 46 (1.52) | 178 (0.83) |
| Policy Maker/Analyst | 7 (0.23) | 105 (0.49) |
| Other | 396(13.09) | 3,134 (14.67) |
| **Affiliation** |  |  |
| Academia | 60 (1.98) | 1,017 (4.76) |
| Organizations of Persons with Disabilities | 83 (2.74) | 503 (2.35) |
| Donor /Funder | 1 (0.03) | 11 (0.05) |
| General health service | 1,047 (34.60) | 4,518 (21.15) |
| Mental health service | 649 (21. 45) | 4,592 (21.50) |
| Ministry of health | 132 (4.36) | 1,416 (6.63) |
| Multilateral organization or development agency | 4 (0.13) | 49 (0.23) |
| Non-Governmental Organizations | 124 (4.10) | 1,486 (6.96) |
| Other Government Ministry/Department/Commission | 66 (2.18) | 654 (3.06) |
| Professional organizations/associations | 232 (7.67) | 1,387 (6.49) |
| Students (Secondary school) | 3 (0.10) | 50 (0.23) |
| Students (University) | 142 (4.69) | 2,087 (9.77) |
| UN organizations and agencies | 9 (0.30) | 96 (0.45) |
| World Health Organization | 19 (0.63) | 283 (1.32) |
| Other | 455 (15.04) | 3,214 (15.04) |

* Missing data (n=37 in the matched sample and n=178 in the pre-training sample) for gender.

** Some participants (n=11 in the matched sample, n=59 in the pre-training sample) reported they reside in a country not registered in the WHO list of countries, and thus they were not classifiable by region or income. WHO regions are abbreviated as follows: AFRO = African Region, AMRO = Region of the Americas, EMRO = Eastern Mediterranean Region, EURO = European Region, SEARO = South-East Asia Region, WPRO = Western Pacific Region

**Supplementary Material 2: Figure showing a map of participants who completed the WHO QualityRights e-training by country (sample, n=42,162; years 2022-2024)**


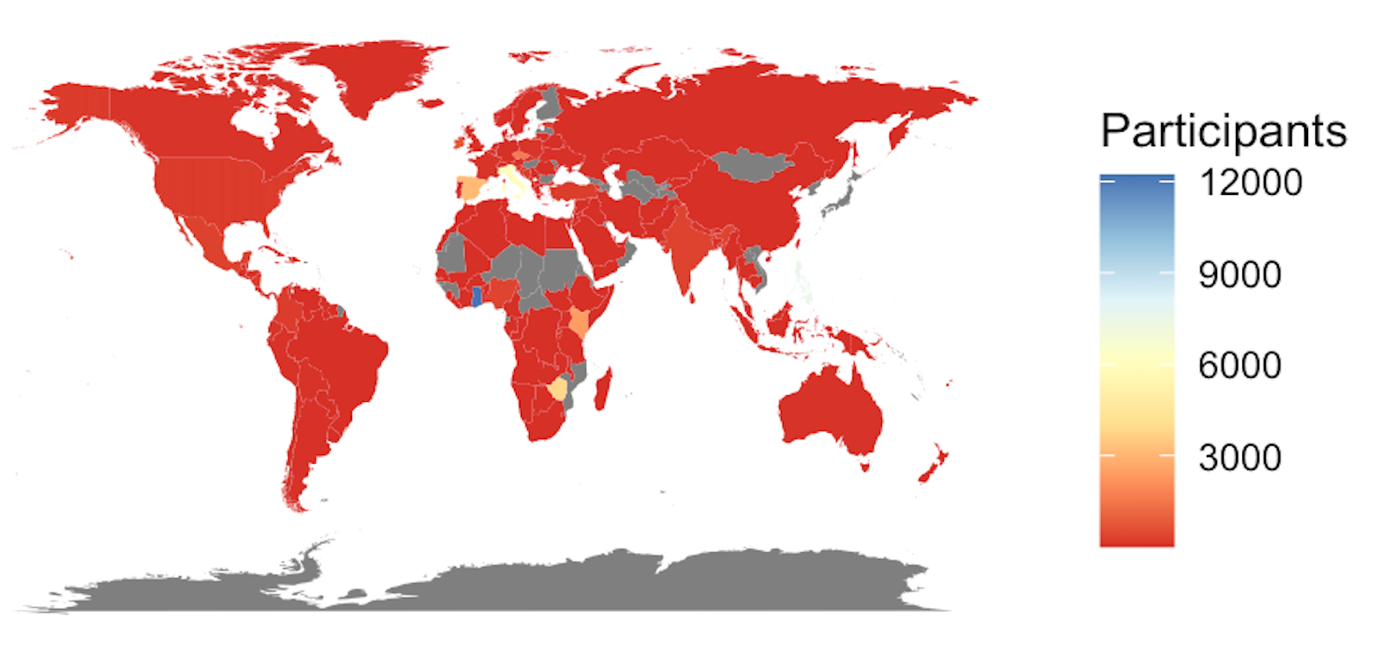


Supplementary Material 3: Table showing changes in mean total scores at the WHO QR Attitudes pre- and post-training (health practitioners, mental health and related practitioners)

|  | **Mean (SD)** | | **Percentage improvement** |
| --- | --- | --- | --- |
|  | **Pre-training** | **Post-training** |  |
| **WHO QR Attitudes Total score** |  |  |  |
| Health practitioners | 45.12 (7.57) | 34.12 (9.61) | 24.38 |
| Mental health or related practitioners | 39.18 (8.77) | 31.22 (9.53) | 20.32 |
|  |  |  |  |

Supplementary Material 4: Table showing inferential statistics (Paired t-test) on average change at the total scores at the WHO QR Attitudes following the e-training (health practitioners, mental health or related practitioners)

|  |  | |  |
| --- | --- | --- | --- |
|  | **Mean difference** | **95% CI** | **d*** |
| **WHO QR Attitudes total score** |  |  |  |
| Health practitioners | 11.01 | 10.53-11.48 | 1.27 |
| Mental health or related practitioners | 7.95 | 7.37-8.54 | 0.87 |
|  |  |  |  |

*Hedge’s g values were nearly identical across all comparisons, with only negligible differences at higher decimal places.

Supplementary Material 5: Table showing median scores pre- and post-training at the WHO QR Attitudes sub-scales (health practitioners, mental health or related practitioners)

|  | **Q1, Median, Q3** | |
| --- | --- | --- |
|  | **Pre-training** | **Post-training** |
| **WHO QR Attitudes Subscales score*** |  |  |
| Subscale 1- Health practitioners | 17, 20, 23 | 13, 16, 19 |
| Subscale 1 - Mental health or related practitioners | 13, 17, 20 | 11, 14, 17 |
| Subscale 2 - Health practitioners | 10, 11, 12 | 6,8,9 |
| Subscale 2 - Mental health or related practitioners | 8, 10, 12 | 5, 7, 9 |
| Subscale 3 - Health practitioners | 12, 14, 16 | 8, 10, 12 |
| Subscale 3 - Mental health or related practitioners | 10, 12, 14 | 7, 9, 11 |
|  |  |  |

* The WHO QR Attitudes scale comprises three subscales: (1) attitudes towards a person-centred recovery-oriented service approach; (2) attitudes towards involuntary and coercive practices; and (3) attitudes towards people with psychosocial disabilities or mental health conditions as decision-makers and full members of society.

Supplementary Material 6: Table showing inferential statistics (Wilcoxon Signed-Rank Test) on change at the sub-scales scores of the WHO QR Attitudes following the e-training (health practitioners, mental health or related practitioners)

|  |  | |
| --- | --- | --- |
|  | **S** | **p-value** |
| **WHO QR Attitudes*** |  |  |
| Subscale 1- Health practitioners | 335782.5 | <.0001 |
| Subscale 1 - Mental health or related practitioners | 75922.5 | <.0001 |
| Subscale 2 - Health practitioners | 376990.5 | <.0001 |
| Subscale 2 - Mental health or related practitioners | 97083 | <.0001 |
| Subscale 3 - Health practitioners | 390397 | <.0001 |
| Subscale 3 - Mental health or related practitioners | 98030 | <.0001 |
|  |  |  |

* The WHO QR Attitudes scale comprises three subscales: (1) attitudes towards a person-centred recovery-oriented service approach; (2) attitudes towards involuntary and coercive practices; and (3) attitudes towards people with psychosocial disabilities or mental health conditions as decision-makers and full members of society.
